# Mechanocatalytic Solvent-Free Esterification of Sugarcane Bagasse

**DOI:** 10.3390/polym10030282

**Published:** 2018-03-08

**Authors:** Qiang Zhang, Xueqin Zhang, Ziyan Zhu, Aiping Zhang, Chunhui Zhang, Xiaoying Wang, Chuanfu Liu

**Affiliations:** 1 State Key Laboratory of Pulp and Paper Engineering, South China University of Technology, Guangzhou 510640, China; zhqiang9@126.com (Q.Z.); xueqin0228@gmail.com (X.Z.); xyw@scut.edu.cn (X.W.); 2 School of Light Industry and Engineering, South China University of Technology, Guangzhou 510640, China; zhuziyan1226@gmail.com; 3 College of Forestry and Landscape Architecture, South China Agricultural University, Guangzhou 510642, China; aiping@scau.edu.cn

**Keywords:** sugarcane bagasse, esterification, ball milling, bio-based materials

## Abstract

Esterification is a versatile way to produce the derivatives of lignocellulose with developed properties. However, the traditional heterogeneous esterification of lignocellulose suffered from the drawbacks of low efficiency, additional reaction medium and heating. In the present study, an efficient method was developed to produce the functionalized sugarcane bagasse (SCB) by ball milling without any additional solvents and heating. The effects of pulverization time, rotation speed, the kind of linear chain anhydrides, the ratio of anhydrides to SCB, with or without pyridine catalyst and the dosage of catalyst were investigated on weight percent gain (*WPG*) of SCB esters. The results indicated that the high efficiency of this mechanocatalystic esterification was probably due to the destroyed crystalline structure and the promoted penetration of the esterifying reagent onto SCB bulk caused by ball milling. The maximum *WPG* of SCB acetate, propionate and butyrate reached 33.3%, 33.6% and 32.4%, respectively. The physicochemical structure of the esterified SCB was characterized with Fourier transform infrared spectra (FT-IR), solid state cross-polarized magic angle spinning ^13^C nuclear magnetic resonance (CP/MAS ^13^C-NMR), X-ray diffraction (XRD), scanning electron microscopy (SEM) and thermogravimetric analysis (TGA). The direct evidence of the esterification occurrence was provided with FT-IR and solid-state CP/MAS ^13^C-NMR. The thermal stability of SCB increased upon the mechanocatalytic esterification. The results implied that the relatively homogeneous modification was achieved with this semi-homogeneous esterification method by ball milling.

## 1. Introduction

The interest in converting biomass to chemicals and materials has increased sharply over the past decade throughout the world from academia to industrial companies [1,2]. Sugarcane bagasse (SCB), a lignocellulosic residue from the sugar industry, is one of the most abundant agro-industrial biomass [3]. However, research activities in the field of SCB were mainly focused on converting it into fuel or using as raw materials in papermaking industry [4,5]. Recently, there are increasing interests towards high value utilization of SCB and various processes have been set up with SCB as raw material [6,7,8].

SCB owns a natural complex supra-molecular structure which primarily composed of lignin (20–30%), cellulose (40–45%) and hemicellulose (30–35%) [9]. Cellulose is a linear homopolysaccharide composed of β-d-glucose units. Hemicellulose is a branched amorphous heterogeneous polysaccharide [10]. Lignin contains a small amount of *p*-hydroxyphenyl (H) residues, in addition of guaiacyl (G) residues and syringyl (S) residues. Cellulose nanofiber is embedded in a complex network of hemicellulose and lignin, which is compared to the structure of reinforced concrete [11]. Many researchers have devoted their efforts to isolating cellulose, lignin and hemicellulose from such a complex structure for bio-based materials [12,13,14]. However, the separation of SCB fractions usually involves complex procedures associated with high energy input, hazardous chemicals and high cost [15]. Producing value-added polymeric materials directly from SCB without separation could provide a facile approach. Esterification is an important way to fabricate products with designable properties from lignocellulosic biomass. Each glucose unit in cellulose carries three hydroxyl groups, locating at the position of C2, C3 (secondary hydroxyl groups) and C6 (primary hydroxyl groups), respectively, which could be substituted by ester groups [16]. Both hemicellulose and lignin also comprise a large amount of available hydroxyl groups for esterification [17]. Previously, many researches have been conducted on the esterification of SCB for different application purposes. For example, thermoformable materials could be synthesized by the esterification of SCB with succinic anhydride, using as an excellent cation exchanger for the adsorption of heavy metal ions, such as Cu^2+^, Ni^2+^, Cr^3+^ and Fe^3+^ [18,19]; oil absorption materials were also prepared using acetic anhydride (AA) as esterifying reagent and the acetylated SCB presented excellent oil sorption capacity, with 1.9 times higher than that of commercial polypropylene oil sorbent [20]; and bioplastic films with mechanical properties compared to cellulose esters were prepared from SCB by esterification and subsequent solution casting [21,22].

The esterification of SCB could be homogeneous or heterogeneous, according to the reaction medium. For homogeneous modifications, dimethyl sulfoxide (DMSO)/tetrabutylammonium fluoride, DMSO/*N*-methylimidazole, DMSO/lithium chloride (LiCl), or ionic liquids could be reaction media, representing an advanced technology with high reaction rate and high substitution [23,24]. However, the DMSO-based solvents are hazardous and difficult to recover [15,25]. Although ionic liquids are green solvents, they are far away from industrial available due to the high cost. The drawbacks of heterogeneous esterification experienced the low efficiency, high reaction temperature as well as the poor properties of derivatives [26]. The low efficiency of heterogeneous esterification could be attributed to the insufficient mass and energy transport between solid phases, which means chemicals could only react with the surface of SCB [27]. It is important for heterogeneous system to maintain intimate contact between chemicals and biomass materials. In the past few years, forming attrition technology by biomass processing facilities such as hammer mills, disk mills or roller mills has been actively researched in this field to overcome the diffusion problem [28,29,30]. However, these methods could not provide a good interaction. Recently, an effective process that effectively “push” the chemicals into contact with the biomass material by planetary ball miller, namely mechanocatalytic reaction, has attracted increasing attention for chemical reaction. Mechanocatalytic process is a solvent-free, environmental-friendly and low cost method, which provides all energy for the reaction by friction, collision and shear interactions [31]. In mechanocatalytic system, all components are dispersed by mechanical impact without any expensive and hazardous solvents. Ball-milling in dry condition has been used to increase the amorphous content of cellulose [32], which makes it possible to enhance reagents penetration during the chemical modification of SCB.

In the present study, SCB esters were prepared through a mechanochemical technique to demonstrate the efficiency of the process. The effects of pyridine catalyst, the kind of linear chain anhydrides, the ratio of anhydrides to SCB, pulverization time and rotation speed were examined on weight percent gain (*WPG*) values. Furthermore, Fourier transform infrared spectra (FT-IR), solid state cross-polarized magic angle spinning ^13^C nuclear magnetic resonance (CP/MAS ^13^C-NMR), X-ray diffraction (XRD), scanning electron microscopy (SEM) and thermogravimetric analysis (TGA) were performed to analyze the structural development of products.

## 2. Materials and Methods

### 2.1. Materials

SCB, obtained from Guangxi Gui-Tang Group Co., Ltd. (Guigang, China), was dried, ground and screened to prepare the 20–35 mesh particles (425–850 μm). The samples were dewaxed with toluene/ethanol (2:1, *v*/*v*) in a Soxhlet extractor for 12 h and then dried at 105 °C for 7 h.

AA was purchased from Kaixin Chemical Reagent Co., Ltd. (Hengyang, China). Propionic anhydride (PA), butyric anhydride (BA) and pyridine were of analytical reagent grade and obtained from Aladdin Reagent Co. (Shanghai, China).

### 2.2. Mechanocatalytic Solvent-Free Esterification of SCB

Oven-dried SCB, esterifying reagents and pyridine were loaded in ZrO_2_ vessels with ZrO_2_ balls and subjected to ball milling for esterification on the planetary ball-miller (Chishun, Nanjing, China). The discontinuous ball-milling, 3 min interval after every 3 min ball-milling, was carried out to control the temperature during the reaction. The specific reaction conditions are listed in Table 1. After the required time, the solid residues were thoroughly washed with 200 mL of ethanol for 5 times to remove unreacted esterifying reagents, pyridine and byproducts. The resulted solids were dried in a cabinet oven at 50 °C for 12 h and further dried at 105 °C for 7 h. Each experiment has been repeated for 3 times. The esterification efficiency was evaluated with *WPG* based on Equation (1):

(1) $$WPG=\frac{M_{1}-M_{0}}{M_{0}}\times 100\%$$

where *M*_0_ and *M*_1_ are the oven-dried weights of the SCB before and after the esterification, respectively.

The degree of substitution (DS) was determined according to the following procedures [22,33]. 0.2 g of sample was weighted accurately and stirred for 30 min in 10 mL of 70% aqueous ethanol. Then 5 mL of 0.5 mol/L of standard NaOH solution was added and stirred for 48 h at 60 °C. The excess alkali was back-titrated with 0.1 mol/L standard HCl solution. Two repetitions of the titration were carried out. The DS was calculated based on Equation (2):

(2) $$\mathrm{DS}=\frac{\left(c_{NaOH}\cdot V_{NaOH}-c_{HCl}\cdot V)-(c_{NaOH}\cdot V_{NaOH}-c_{HCl}\cdot V_{c}\right)}{m-[(c_{NaOH}\cdot V_{NaOH}-c_{HCl}\cdot V)-(c_{NaOH}\cdot V_{NaOH}-c_{HCl}\cdot V_{c})]\cdot M\times 10^{-3}}$$

where *c_NaOH_* is the molarity of standard NaOH solution, *V_NaOH_* is the volume of standard NaOH solution, *c_HCl_* is the molarity of standard HCl solution, *V* and *V_c_* are the volume of standard HCl solution added into SCB ester samples and into the control sample, respectively, *m* is the weight of sample analyzed and *M* is the net increase in the molar mass of each hydroxyl substituted with the acyl residue.

### 2.3. Characterization of the Esterified SCB

FT-IR spectra of the samples were recorded on a Bruker FT-IR spectrophotometer (Karlsruhe, Germany) with a KBr disc containing 1% finely ground samples in the range of 4000–400 cm^−1^ at a resolution of 4 cm^−1^.

The solid-state CP/MAS ^13^C-NMR spectra were recorded on a Bruker DRX-400 spectrometer (Karlsruhe, Germany). The detailed parameters for CP/MAS ^13^C-NMR analysis were listed as follows: number of scans, 2170; receiver gain, 2050; acquisition time, 0.339 s; relaxation delay, 1.0 s; pulse width, 3.16 s; spectrometer frequency, 100.61 MHz; and spectral width, 30,214.9 Hz.

XRD patterns were collected on a Bruker D8 Advance X-ray diffractometer (Karlsruhe, Germany) with a Ni filter and Cu-K_α_ radiation (λ = 0.15418 nm). The diffraction patterns were measured with a scanning speed of 0.0625°/min at 40 kV and 40 mA. To exhibit the changes of crystalline structure of cellulose in SCB upon the mechanocatalytic esterification, the crystallinity index (*CrI*) was determined according to Equation (3) [34]:

(3) $$CrI=\frac{I_{002}-I_{am}}{I_{002}}$$

where *I*_002_ is the peak intensity of the (002) plane (2θ = 22.5°) and *I_am_* is the peak intensity of amorphous scatter (2θ = 18°).

The morphology changes of SCB upon the esterification by ball milling were observed using a ZEISS Merlin SEM (Jena, Germany). Prior to the measurement, a thin layer of Pd–Au alloy was coated on the samples.

TGA was performed on a TA Q500 analyzer (New Castle, DE, USA) to obtain the thermal stability of the samples. The apparatus was continually flushed with nitrogen. Samples weighed approximately 10 mg were heated in an aluminum crucible from room temperature to 600 °C at a heating rate of 10 °C/min.

## 3. Results and Discussion

### 3.1. Preparation of Esterified SCB

**Scheme 1:**
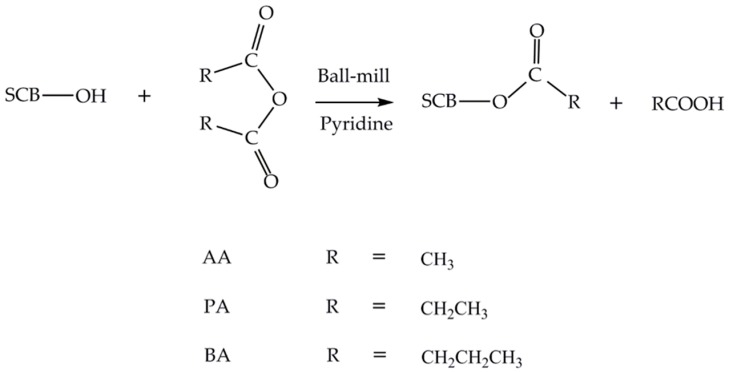
Mechanocatalytic esterification of SCB with AA, PA or BA during ball milling at the presence of pyridine.

Esterification is one of the most effective methods to enhance a variety of valuable properties of lignocellulosic materials. In the present study, the modification of SCB was performed with linear chain anhydride AA, PA, or BA as esterifying reagent by ball milling. Keeping AA/SCB ratio at 11:1 mmol/g, the molar ratio of AA to pyridine at 1:1, rotation speed of ball-milling at 800 r/min and without additional solvent and heating, the increase of pulverization time from 0.5 to 4 h resulted in an increment in the *WPG* of SCB acetate from 10.7% to 22.2%. This might be attributed to the further destruction of the cellulose crystalline structure of SCB during ball milling. By contrast, the control sample was obtained at 800 r/min for 4 h without the addition of anhydrides and pyridine and the *WPG* of the control sample was only −5.0%. As shown in Table 1, a similar trend between DS and *WPG* was observed. These results were parallel with the result reported before [22], suggesting that it was satisfactory for characterizing the efficiency of the machanocatalytic esterification with *WPG*. The difference of *WPG* and DS values between SCB acetates and the control sample indicated that SCB was esterified by ball milling under the selected conditions and this mechanocatalystic solvent-free reaction is shown in Scheme 1.

The effects of reaction conditions, including rotation speed, the ratio of anhydride to SCB, the ratio of catalyst pyridine to anhydride and different anhydrides as esterifying reagents on the *WPG* of SCB esters were investigated and the results are listed in Table 1. The *WPG* of SCB acetates gradually increased from 22.2% to 27.3% with the increased rotation speed of ball-milling from 800 to 1200 r/min This result suggested that the efficiency of modification was improved with input dynamic energies which produced by the interaction between frictional and impact forces during ball-milling without any external heating. Furthermore, the friction resulted from ball milling led to better mixing of SCB, anhydrides and pyridine which enhanced the reactivity of the reactants. Meanwhile, the size reduction and the destruction of crystalline structure resulted in the increasing disordered accessible regions of SCB and more active hydroxyl groups which could further increase the reaction efficiency. The theoretical calculations indicated that the content of total hydroxyl groups in lignocellulosic biomass was about 14.9 mmol/g [35]. The molar ratio of anhydrides to hydroxyl groups in the mechanocatalytic esterification (Scheme 1) is 1:1. Therefore, the ratio of anhydride to SCB was selected from insufficient to overdose in the range of 3:1 to 32:1 mmol/g to investigate the effect on the mechanocatalytic esterification. The *WPG* of SCB acetates was very low (<7.0%) and irregular without addition of catalyst pyridine, implying only slight acetylation even at high dosage of AA in the absence of pyridine; while *WPG* significantly increased under the same conditions at the presence of pyridine. These results indicated that acetylation of SCB easily occurred during ball milling at the presence of pyridine. The increase of AA/SCB ratio from 3:1 to 27:1 mmol/g resulted in a substantial improvement of *WPG* from 7.5% to 33.3%, indicating pyridine was the effective and high efficient catalyst for SCB acetylation during ball milling; whereas the further increase of AA/SCB ratio from 27:1 to 32:1 mmol/g led to a slight decrease in *WPG* from 33.3% to 31.8%. Similarly, when the ratio of esterifying reagents (PA or BA) to SCB increased from 4:1 to 23:1, the *WPG* of SCB propionates and butyrates increased from 21.2% to 33.6% and 12.5% to 32.4%, respectively, while it decreased from 33.6% to 31.3% and 32.4% to 30.9%, respectively, with the further increase of the esterifying reagents/SCB ratio from 23:1 to 32:1. These decreases were probably due to the increased degradation of SCB at the presence of excess acidic anhydrides and the byproduct acid [20,36,37]. Due to the limited accessibility of the heterogeneous reaction, the *WPG* of the esterified plant cell walls obtained from the common esterification methods rarely exceeds 25% [35]. In the present study, the maximum *WPG* of 33.3%, 33.6% and 32.4% was achieved for SCB acetate, propionate and butyrate, respectively, by ball milling at 1200 r/min for 4 h. The high efficiency of this mechnocatalytic esterification of SCB was probably due to the destroyed crystalline structure and the promoted penetration of the esterifying reagent onto SCB bulk caused by ball milling.

### 3.2. FT-IR

**Figure 1:**
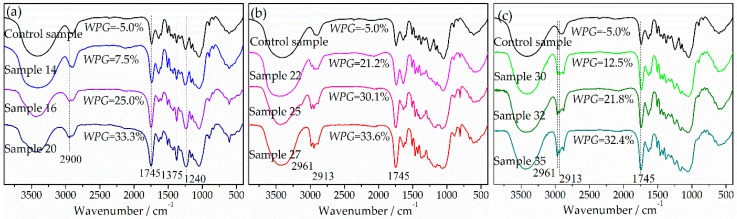
FT-IR spectra of the control sample and SCB acetates (**a**), propionates (**b**) and butyrates (**c**) obtained by the mechanocatalytic esterification.

FT-IR spectra of control sample and SCB acetate, propionate and butyrate with different *WPG* are illustrated in Figure 1. For a better comparability, the FT-IR spectra has been normalized to the band at 1047 cm^−1^ for glycosidic linkage (C–O–C) of cellulose and hemicelluloses [38]. According to the reported literatures [39,40], the characteristic bands were assigned. The bands at 2900 cm^−1^ for C–H stretching, 1745 cm^−1^ for C=O stretching, 1375 cm^−1^ for C–H bending in –CH_3_ and 1240 cm^−1^ for C–O stretching in ester became stronger in SCB acetate compared with those in the control sample (Figure 1a), which was probably due to the introduced acetyl groups. The intensities of the four bands were also improved with the increased *WPG*. These results confirmed the occurrence of acetylation by mechanocatalytic modification under the selected conditions. The absence of band at 1760–1840 cm^−1^ and 1700 cm^−1^ which could be attributed to the characteristic C=O band of anhydrides and carboxylic acid confirmed that the unreacted anhydrides and byproduct had been removed completely [36]. In Figure 1b, the two new band appeared at 2913 and 2961 cm^−1^ in SCB propionate, probably associated with the asymmetric stretching of methyl and methylene group, respectively. In addition, the intensity of the absorbance at 1745 cm^−1^ for C=O stretching significantly increased with the improved *WPG* of SCB propionates. These results provided the evidences of the occurrence of the esterification between SCB and PA by ball milling under the selected conditions and the introduction of acetyl groups onto SCB. The similar results were also observed in SCB butyrates (Figure 1c). These results demonstrated the success of mechanocatalytic SCB esterification without any additional solvents by ball milling.

### 3.3. CP/MAS ^13^C-NMR

**Figure 2:**
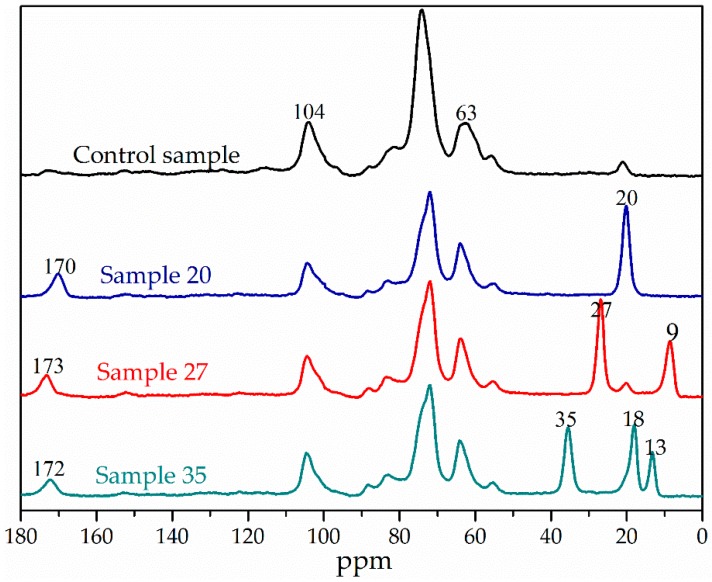
Solid state CP/MAS ^13^C-NMR spectra of the control sample and SCB acetate (sample 20), propionate (sample 27) and butyrate (sample 35).

To further confirm the occurrence of SCB acylation, the solid state CP/MAS ^13^C-NMR spectra of the control sample and SCB acetate (sample 20), propionate (sample 27) and butyrate (sample 35) were collected, as shown in Figure 2. Compared with the spectrum of the control sample, a new carbon signal appeared at around 173 ppm for carbonyl group in those of SCB acetate, propionate and butyrate, indicating the occurrence of esterification by ball milling under the selected conditions. In addition, the intensity of the signal at 20 ppm for methyl increased significantly in SCB acetate, demonstrating the introduction of acetyl group onto SCB. Another two new carbon signals at 9 and 27 ppm appeared in SCB propionate, probably originated from the carbon in methyl and methylene group, implying the attachment of propionyl group onto SCB. Similarly, another three new carbonyl signals were present at 13, 18 and 35 ppm in SCB butyrate, providing the evidence of the attachment of butyryl group onto SCB. There results further confirmed the occurrence of SCB esterification during ball milling, which was consistent with the results from FT-IR. In addition, there was no noticeable changes on the primary carbon signals from polysaccharides including 104 (C-1) and 63 ppm (cellulose C-6) [41], indicating the primary structure unchanged after the mechanocatalytic esterification by ball milling.

### 3.4. XRD

**Figure 3:**
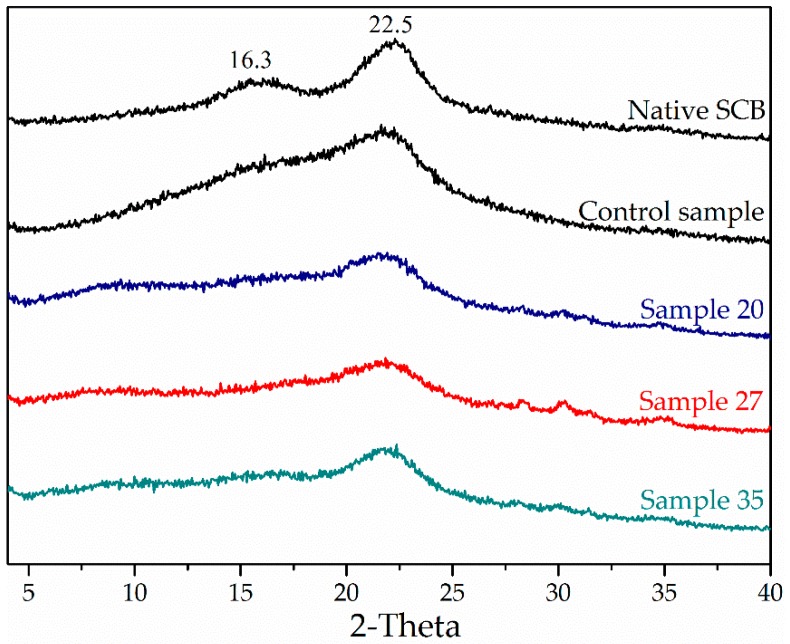
XRD patterns of native SCB, the control sample and SCB acetate (sample 20), propionate (sample 27) and butyrate (sample 35).

To evaluate the changes of crystalline structure upon ball milling and the mechanocatalytic esterification of SCB, the XRD patterns of native SCB, the control sample and SCB acetate (sample 20), propionate (sample 27) and butyrate (sample 35) were collected, as illustrated in Figure 3.

**Figure 4:**
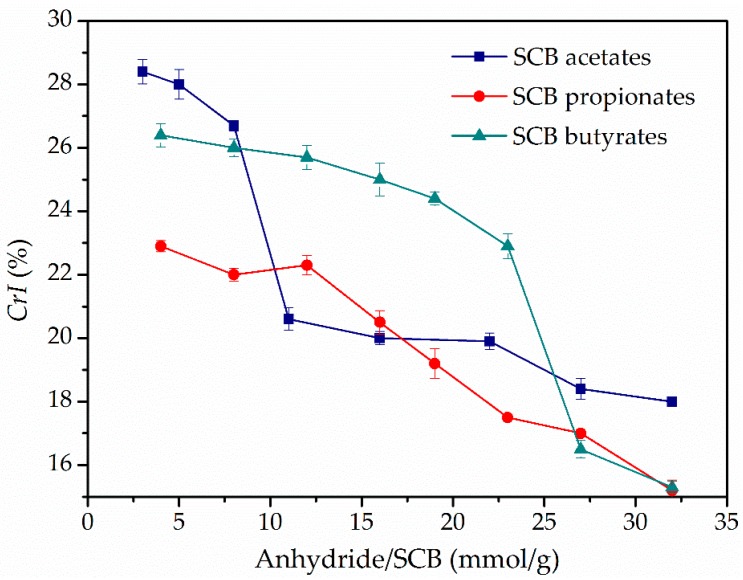
The *CrI* values of SCB acetates, propionates and butyrates at different anhydride/SCB ratio.

Native SCB exhibited a typical cellulose I pattern with two bands at 16.3° for (10$\bar{1}$) plane and 22.5° for (002) plane, respectively [42]. Ball milling remarkably destroyed the native crystalline structure of cellulose in SCB when compared with native SCB and the control sample. This de-crystallization was confirmed by the disappearance of 10$\bar{1}$ plane, therefore, the penetration of anhydrides into SCB increased in the following esterification with AA, PA and BA in comparison with the common heterogeneous esterification. The promoted accessibility of hydroxyl functionality of SCB caused by ball milling guaranteed the successful mechanocatalytic esterification. The *CrI* was estimated from XRD curves and the *CrI* changes of SCB esters at different anhydride/SCB ratio is illustrated in Figure 4. Clearly, the increase of anhydride/SCB ratio led to the improved de-crystallization under the selected conditions. At high anhydride/SCB ratio, the low *CrI* and the increased *WPG* were simultaneously obtained, indicating that the increased esterification was maybe helpful for de-crystallization at the presence of by-product acid produced upon esterification. However, the random distribution of substitution groups could lead to a slightly increase of *CrI* values [43]. The slighter slope of SCB butyrates might result from the regular chain packing. The lowest *CrI* values of SCB acetate, propionate and butyrate were 18.0 ± 0.12%, 15.2 ± 0.33% and 15.3 ± 0.19%, respectively, at anhydride/SCB ratio of 32:1 mmol/g, which was mainly due to the severe acid degradation.

### 3.5. SEM

**Figure 5:**
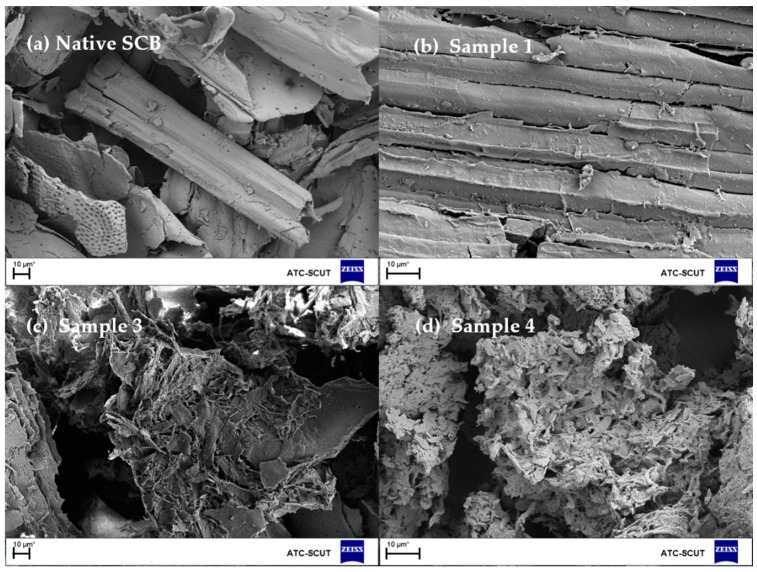
SEM images of native SCB (**a**) and SCB acetate obtained for 0.5 h (sample 1, **b**), 2 h (sample 3, **c**), 4 h (sample 4, **d**).

To elucidate the changes of surface morphology upon the mechanocatalytic esterification, Figure 5 lists the SEM images of native SCB and SCB acetate samples 1 (0.5 h), 3 (2 h) and 4 (4 h). Clearly, native SCB exhibited smooth surface (Figure 5a). The surface was partly damaged upon ball milling and esterification for 0.5 h and the fibrous structure could still be observed (Figure 5b). Prolonging ball milling and esterification time to 2 h, native structure of SCB was significantly destroyed and much more linear acetyl group was attached, resulting in the noticeable small pieces from rod-like shape (Figure 5c). Further improving to 4 h, the native SCB structure almost totally destroyed and converted to the rough and agglomerated fragments (Figure 5d). Therefore, the mechanocatalytic esterification of SCB seemed to achieve the relatively homogeneous modification as the semi-homogeneous methods.

### 3.6. Thermal Stability

**Figure 6:**
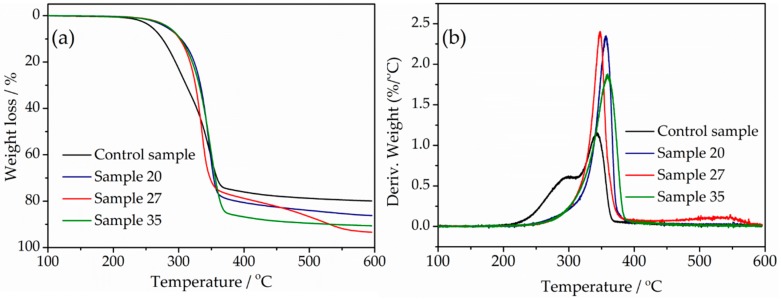
TGA and DTG curves of the control sample and SCB acetate (sample 20), propionate (sample 27) and butyrate (sample 35).

The thermal stability of control sample and SCB ester samples was examined by TGA in N_2_ atmosphere. The TGA and DTG curves of the control sample and SCB acetate (sample 20), propionate (sample 27) and butyrate (sample 35) are shown in Figure 6.

The weight loss below 100 °C was mainly attributed to the evaporation of water which could be influenced by different storage conditions [36]. Thus, the weight loss in the range of 100 °C to 600 °C of control sample and SCB esters were used to investigate the effect of acylation with AA, PA and BA on the thermal behavior of SCB. The control sample began to decompose at 214 °C, while SCB esters started to decompose at 250–255 °C (Figure 6a). As shown in DTG curves (Figure 6b), the maximum degradation rate of appeared at higher temperature that that of control sample. These increasing trends of decomposition temperature of the esterified SCB implied that the thermal stability of SCB increased upon the mechanocatalytic esterification by ball milling. The similar increased thermal stability was reported in the previous literatures [43,44,45,46]. The char formation during thermal pyrolysis is resulted from complex dehydration reactions. The higher thermal stability of SCB esters might due to the reduced content of available hydroxyl groups which would lead to a weakness of the dehydration process [47]. The changes of the thermal stability also suggested the esterification occurred by ball milling under the selected conditions. The higher thermal stability of SCB esters obtained with the mechanocatalytic esterification than the control sample [48], indicating the reaction between SCB and anhydrides does occur by ball milling under the selected conditions, which was parallel to the results from FT-IR and solid state CP/MAS ^13^C-NMR analyses.

## 4. Conclusions

SCB esters were directly prepared by ball milling with AA, PA and BA as liner chain esterifying reagents, respectively, without additional solvent and heating. The esterification efficiency could be significantly improved at the presence of pyridine. The high efficiency of this mechanocatalystic esterification was probably due to the destroyed crystalline structure and the promoted penetration of the esterifying reagent onto SCB bulk caused by ball milling. The maximum *WPG* of SCB acetate, propionate and butyrate reached 33.3%, 33.6% and 32.4%, respectively. FT-IR, solid state CP/MAS ^13^C-NMR, XRD, SEM and TGA provided the evidence of the occurrence of esterification. The attachment of new esterified groups was directly confirmed with FT-IR and solid-state CP/MAS ^13^C-NMR. The thermal stability of SCB increased upon the mechanocatalytic esterification. This semi-homogeneous esterification method could achieve similar efficiency to homogeneous modification.

## Figures and Tables

**Table 1:** *WPG* and DS values of SCB esters obtained by ball milling under various conditions.

No.	Anhydride	Anhydride/SCB (mmol/g)	Pyridine/Anhydride (mol/mol)	Rotation Speed (r/min)	Time (h)	*WPG* (%)	DS (mmol/g)
Control	/	/	/	800	4.0	−5.0 ± 0.13	/
1	AA	11:1	1:1	800	0.5	10.7 ± 0.21	4.9 ± 0.51
2	AA	11:1	1:1	800	1.0	16.5 ± 0.47	5.5 ± 0.34
3	AA	11:1	1:1	800	2.0	17.8 ± 0.35	5.8 ± 0.41
4	AA	11:1	1:1	800	4.0	22.2 ± 0.64	7.6 ± 0.39
5	AA	11:1	1:1	1000	4.0	24.1 ± 0.32	7.8 ± 0.22
6	AA	3:1	/	1200	4.0	2.5 ± 0.21	3.0 ± 0.42
7	AA	5:1	/	1200	4.0	4.6 ± 0.34	3.5 ± 0.19
8	AA	8:1	/	1200	4.0	6.4 ± 0.27	3.7 ± 0.21
9	AA	11:1	/	1200	4.0	5.6 ± 0.49	3.8 ± 0.53
10	AA	16:1	/	1200	4.0	1.9 ± 0.21	2.7 ± 0.21
11	AA	22:1	/	1200	4.0	3.2 ± 0.52	3.5 ± 0.36
12	AA	27:1	/	1200	4.0	4.2 ± 0.46	3.6 ± 0.41
13	AA	32:1	/	1200	4.0	5.3 ± 0.39	3.9 ± 0.39
14	AA	3:1	1:1	1200	4.0	7.5 ± 0.62	4.1 ± 0.27
15	AA	5:1	1:1	1200	4.0	18.1 ± 0.58	6.2 ± 0.32
16	AA	8:1	1:1	1200	4.0	25.0 ± 0.47	8.1 ± 0.54
17	AA	11:1	1:1	1200	4.0	27.3 ± 0.72	8.4 ± 0.31
18	AA	16:1	1:1	1200	4.0	30.3 ± 0.43	8.3 ± 0.48
19	AA	22:1	1:1	1200	4.0	30.0 ± 0.67	8.6 ± 0.34
20	AA	27:1	1:1	1200	4.0	33.3 ± 0.36	8.9 ± 0.52
21	AA	32:1	1:1	1200	4.0	31.8 ± 0.68	8.6 ± 0.29
22	PA	4:1	1:1	1200	4.0	21.2 ± 0.52	5.3 ± 0.41
23	PA	8:1	1:1	1200	4.0	24.2 ± 0.44	6.4 ± 0.37
24	PA	12:1	1:1	1200	4.0	25.8 ± 0.51	6.6 ± 0.16
25	PA	16:1	1:1	1200	4.0	30.1 ± 0.82	6.8 ± 0.32
26	PA	19:1	1:1	1200	4.0	30.2 ± 0.49	6.6 ± 0.42
27	PA	23:1	1:1	1200	4.0	33.6 ± 0.79	7.9 ± 0.22
28	PA	27:1	1:1	1200	4.0	31.7 ± 0.41	7.2 ± 0.29
29	PA	32:1	1:1	1200	4.0	31.3 ± 0.61	7.0 ± 0.33
30	BA	4:1	1:1	1200	4.0	12.5 ± 0.36	2.1 ± 0.29
31	BA	8:1	1:1	1200	4.0	17.2 ± 0.52	2.4 ± 0.41
32	BA	12:1	1:1	1200	4.0	21.8 ± 0.29	2.9 ± 0.53
33	BA	16:1	1:1	1200	4.0	23.7 ± 0.42	3.3 ± 0.17
34	BA	19:1	1:1	1200	4.0	27.1 ± 0.51	3.9 ± 0.23
35	BA	23:1	1:1	1200	4.0	32.4 ± 0.67	4.6 ± 0.27
36	BA	27:1	1:1	1200	4.0	30.1 ± 0.46	4.1 ± 0.42
37	BA	32:1	1:1	1200	4.0	30.9 ± 0.51	4.3 ± 0.15
